# The Interactive Influence of Perceived Ownership and Perceived Choosership of Stocks on Brain Response to Stock Outcomes

**DOI:** 10.3389/fpsyg.2017.00008

**Published:** 2017-01-30

**Authors:** Zhe Shang, Lei Wang, Han Wu

**Affiliations:** School of Psychological and Cognitive Sciences and Beijing Key Laboratory of Behavior and Mental Health, TAETEA Consumer Research Center, Peking UniversityBeijing, China

**Keywords:** behavioral social neuro-finance, choosership effect, ownership effect, choice effect, feedback-related negativity (FRN), emotion, responsibility, neural economics

## Abstract

The present research examined the influence of perceived ownership (self/other) and perceived chooser (self/other) of stocks on brain activity, and investigated whether differential brain responses to stock outcomes as a result of perceived differences in ownership of stock would be modulated by perceived chooser of stock. We used a 2 (stock chooser: self, other) × 2 (stock owner: self, other) within-subject design to represent four types of chooser-owner relationships. Brain potentials were recorded while participants observed increasing and decreasing stock prices. Results showed that observations of stock outcomes among four types of chooser-owner relationships elicited differentiated feedback-related negativity (d-FRN: differences in FRN waves between losses and gains, reflecting violations of expectancy to stock outcomes): (1) Self-chosen-other-owned stocks evoked significantly larger d-FRN discrepancies than self-chosen-self-owned stocks, indicating a greater expectancy violation to others' losses than to one's own, demonstrating a *reversed ownership effect*. Moreover, people high in conscientiousness showed an increase in this trend, suggesting a stronger other-consideration; (2) Self-chosen-self-owned stocks and other-chosen-self-owned stocks revealed no significant d-FRN discrepancy, showing no *choosership effect* beyond the *ownership effect*; (3) Other-chosen-self-owned stocks evoked a significantly stronger d-FRN discrepancy than other-chosen-other-owned stocks, demonstrating an *ownership effect*; (4) Self-chosen-other-owned stocks evoked a significantly stronger d-FRN discrepancy than other-chosen-other-owned stocks, revealing a *choosership effect*. These findings suggest that the ownership effect could be reversed by conscientiousness induced by perceived choosership in the agency relationship, while the choosership effect is attenuated and even disappears under the influence of perceived ownership.

## Introduction

People may take on diverse financial roles in the stock market: observers, buyers, fund managers, or clients. These four roles can be classified according to two categories: ownership (the individual who owns the stock) and choosership (the individual who selected the stock). The roles concerning ownership (self-owned stocks vs. other-owned stocks) and the roles concerning choosership (self-chosen stocks vs. other-chosen stocks) are combined to create four chooser-owner relationships. Intriguingly, people may take on all four financial roles simultaneously. How ownership and choosership of stocks individually and interactively influence brain activities while observing stock outcomes (gains or losses) across the four roles remains unclear. The present study aimed to investigate the individual and interactive influence of perceived ownership and perceived choosership of stocks on stock outcome evaluations.

It has been found that ownership and choosership are two factors that influence preference of objects (Huang et al., 2009). People tend to prefer their own possessions to those they do not own, a phenomenon termed ownership effect (Beggan, 1992; Nesselroade et al., 1999). The choosership effect, which is known as the post-decisional spreading of alternatives, reveals that for the two self-own objects (one self-chosen and one other-chosen), people would more like the self-chosen one rather than the other-chosen one, even if the choosership is only imagined (Huang et al., 2009).

Ownership effect is the tendency to overvalue self-owned possessions to maintain a positive self-image due to a self-enhancement motivation (Belk, 1988; Beggan, 1992; Nesselroade et al., 1999). This bias can appear in various social-cultural contexts. An example lies in the favorable evaluation of objects, where ownership has been hypothesized to increase the attractiveness of objects (Heider, 1958). This effect has been observed with participants rating their own objects more favorably than others' objects, both in studies where there was actual ownership of objects (Beggan, 1992; Nesselroade et al., 1999), and also in studies without real ownership (Sen and Johnson, 1997; De Dreu and van Knippenberg, 2005; Huang et al., 2009).

The effect of choosership on outcome evaluation has been studied extensively over several decades and has been deemed as both desirable and powerful (Iyengar and Lepper, 1999). When people choose between two alternatives that are similarly attractive, they evaluate the chosen alternative substantially more positively than the other options, so as to reassert their autonomy (Brehm, 1966). As for object evaluation, like object preference, a choosership effect has been proposed and termed as the phenomenon where choosership itself is powerful enough to induce liking, even in the condition where choosing is an illusion and does not actually occur (Huang et al., 2009). However, unlike in object evaluations where objects often hold neutral valence, the effect of choosership on distinctive positive (gains) and negative (losses) outcomes has seldom been studied under an imagined condition without real choice processes.

The present research aimed to study the influence of perceived ownership and perceived choosership on stock outcome evaluations by recording brain activities, and also investigate whether differences in brain activity as a response to differential ownership would be modulated by choosership. To this end, we investigated the individual and interactive influence of perceived ownership and perceived choosership on stock outcome evaluations by developing a 2 (stock chooser: self, other) × 2 (stock owner: self, other) × 2 (stock outcome: gain, loss) within-subject design. By doing so, we developed a special situation that combines both *the owner* (self-owned stock vs. other-owned stock) and *the chooser* (self-chosen stock vs. other-chosen stock) to mimic four financial roles in the stock market. Each role represents one of four financial roles: (1) a buyer, who purchases a stock that he has chosen for himself (or self-chosen, self-owned stock, abbreviated as SCSO), acting as an independent individual investor; (2) a broker, who is involved with a stock that he has chosen for another individual (or self-chosen, other-owned stock, SCOO), acting as an agent (e.g., fund manager) who selects stocks for clients but does not own the stocks; (3) a client, involved in a stock chosen by an agent (or other-chosen, self-owned stock, OCSO), acting as a fund holder who owns the stocks but delegates the selection of stocks to other people; (4) an observer, involved in a stock that another person chooses for him/herself (or other-chosen, other-owned stock, OCOO), acting as an onlooker or observer. As for outcome comparison, we compared two of the four financial roles each time. We further compared the outcome evaluation of each pair of financial roles and inferred the possible emotional differences in each comparison group.

When applying electrophysiological methods, converging evidence implies that feedback related negativity responses (FRNs) reflects the neural mechanisms underlying the evaluation of one's own losses and gains (Yeung and Sanfey, 2004; Hajcak et al., 2005, 2007; Sato et al., 2005; Toyomaki and Murohashi, 2005; Yeung et al., 2005; Yu et al., 2007; Luu et al., 2009). FRN is elicited by the incongruity between an actual outcome (e.g., monetary losses) and an initial expectancy (e.g., monetary gains), reflecting the expectancy violation in outcome evaluation tasks (Yu and Zhou, 2006; Itagaki and Katayama, 2008; Kang et al., 2010; Leng and Zhou, 2010; Marco-Pallarés et al., 2010; Ma et al., 2011). FRN is distributed over the fronto-central recording sites and reaches a maximum at ~200–300 ms after the onset of the outcome feedback. FRN is more pronounced for negative feedback associated with unfavorable outcomes, such as incorrect responses or monetary losses, than for positive feedback (Miltner et al., 1997; Gehring and Willoughby, 2002; Müller et al., 2005; Yu and Zhou, 2009). More specifically, FRN reflects a fast good–bad evaluation of feedback, with its amplitude depending on the relationship between the actual and the expected outcomes (Holroyd and Coles, 2002; Nieuwenhuis et al., 2004). In addition, Gehring and Willoughby (2002) argued that FRN (referred to as the MFN) reflects an evaluation of the affective or motivational significance of errors detected by cognitive monitoring processes. This suggests that we can use FRN to speculate on the emotional intensity that may be hidden behind an outcome evaluation. Additionally, to minimize the effects of overlap of FRN with negative ERP components, we calculated the difference of FRN waveforms by subtracting the ERPs elicited by gain from the ERPs elicited by loss, following the methods of Holroyd and Krigolson (2007), and termed the FRN difference between losses and gains d-FRN.

The current research aimed to investigate how individuals respond to financial outcomes in different person-stock relationships. We proposed a new scenario-simulation paradigm to assign four financial roles by priming both ownership and choosership, which can compare the processing of outcome evaluation reflected by FRN when assigned to each of the four different roles. We focused on a financial context in which the “other” and the “self” are not simply friends or strangers; instead, they are bound together by profit-based relationships. This helps further understand the functional significance of FRN involved across multiple financial roles, and by doing so, contributes to the new research area of *social*-*neuro-finance*.

## Materials and methods

### Participants

A total of 22 healthy students (9 males, 13 females; mean age ± SD = 21.40 ± 1.73 years) from different universities voluntarily participated in the study. All participants were right-handed with normal or corrected-to-normal vision. No participants with chronic diseases, mental disorders, medication, or those smoked or abused alcohol were recruited for the experiment.

The experiment was conducted in accordance with The Code of Ethics of the World Medical Association (Declaration of Helsinki). Participants were given written instructions before the experiment began. Each participant knew that at the end of the experiment, they would be reimbursed for their time with US $15. We used a fixed payment since subjects didn't make any operations that changed the results in stock exchange.

### Design and procedures

**Figure 1:**
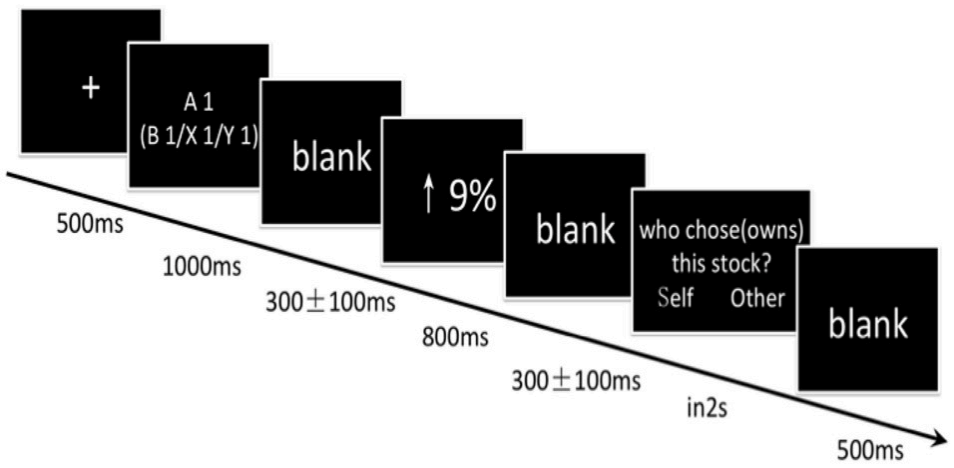
**Sequence of events in a single trial**. Each trial began with the presentation of a fixation cross at the center of the screen for 500 ms against a black background. Then the stock name of one of the twelve memorized stocks (e.g., A1) was presented for 1000 ms. After a jittered blank interval of 200, 300, or 400 ms, a ratio of stock price increase or decrease by 3, 6, or 9% (white and Song font, size 32) was presented to indicate the movement of stock price. The ratio was presented for 800 ms. Participants' EEG signals from −200 to 800 ms of this screen were extracted for analyses. The ratio was followed by another jittered blank interval of 200, 300, or 400ms. At the end of each trial, one of the following two questions, “Who chose the stock (A1),” or “Who owns the stock (A1)” appeared as the “question” screen. The two answer options: the “self” and the “other” (in words), was randomly presented on the left or right side of the “question” screen. The participants were asked to judge the owner or the chooser of the stock by pressing a corresponding key (n, m) as quickly as possible within 2 s. The “question” screen did not disappear until participants responded. The last screen in the trial was blank and lasted for 500 ms.

The experiment adopted a 2 × 2 × 2 within-subjects design, with the choosership of the stocks (self-chosen, other-chosen), the ownership of the stocks (self-owned, other-owned), and the stock outcome (gain, loss) as the three within-subject factors. We used the combination of choosership and ownership to set up four person-stock relationships in which participants played four financial roles. In the four person-stock relationships, the corresponding four types of stocks were named A/B/X/Y (shown in Figure 1). We asked participants to view ratios of stock price change (e.g., rise or fall with a 3, 6, and 9% ratio) and then asked them to recall whether the stocks belonged to the self (the participants themselves) or the other, and whether the stocks were chosen by the self or the other. Participants' event-related potentials (ERPs) evoked by the presentation of the outcome (i.e., the rise and fall of stocks) were recorded while they occupied different financial roles. It is worth noting that in the present research, the paradigm allowed participants to experience different roles across trials within one session.

Participants were told that the experiment consisted of three parts: a practice task, a main experiment task, and a questionnaire task. In the practice part, participants completed a 5-min training session prior to the main experiment. After the main experiment, participants were asked to report their feelings.

In the main experiment task, participants read the following two-page scenarios on paper:

(Page 1)

Welcome to this experiment!

Please visualize the following scenario carefully and answer the following questions:

*Recently people pay more and more attention to financial investment and want to gain more profits. There are different types of financial products, such as stocks, funds, bonds etc. High return always comes with high risk*.

Please imagine the following scenes when you buy a financial product:

*Financial role 1: You choose 3 stocks in the stock market and have decided to buy them*.

*Denote them as A1, A2, and A3*.

*Remember that you made the decision for yourself and bought them by yourself*.

*Financial role 2: Someone named Wang wanted you to help choose some stocks. You chose 3 stocks for him to buy*.

*Denote the 3 stocks as B1, B2, and B3*.

*Remember that you made the decision for Wang and he bought (and owned) the stocks*.

*Financial role 3: You asked someone named Li to choose 3 stocks for you. Then you bought the stocks*.

*Denote them as X1, X2, and X3*.

*Remember that Li made the decision for you and you bought (owned) the stocks*.

*Financial role 4: Someone named Zhang chooses 3 stocks and bought them by him/herself (in accordance with the participant's gender)*.

*Denote them as Y1, Y2, and Y3*.

*Remember that Zhang made the decision and bought (owned) the stocks him/herself*.

*Note: The initial investments are all 100,000 dollars. People who buy the stocks will suffer from the loss or profit from the gain*.

*Please take 2 min to remember these codes for stocks in the four scenarios. They will be used in the following experiment*.

Please answer the following questions:

1. The codes of stocks, which you made the decision about and you bought, are:

___ __ ___

2. The codes of stocks, which you made the decision about and another person bought, are:

___ __ ___

3. The codes of stocks, which another person made the decision about and they bought, are:

___ __ ___

4. The codes of stocks, which another person made the decision about and you bought, are:

___ __ ___

(Page2)

To make sure that the participants had memorized which stocks belonged to them and which to the “other,” they were asked to write down on a blank paper the stocks' names in each scenario before the EEG recording. Altogether, there were 12 stocks (A1, A2, A3, B1, B2, B3, X1, X2, X3, Y1, Y2, Y3) divided into four person-stock relationships. For control purposes, participants were further instructed that all stocks started at the same price.

Each trial began with the presentation of a fixation cross at the center of the screen for 500 ms against a black background. Then the name of one of the twelve memorized stocks (e.g., A1) was presented for 1000 ms. During the experiment, we asked participants to view ratios of stock price changes. After a jittered blank interval of 200, 300, or 400 ms, a ratio of stock rose or declined by 3, 6, or 9% (white and Song font, size 32) could be used to describe the change of previous stocks. The ratio was presented for 800 ms. Participants' EEG signals from −200 to 800 ms of this screen were extracted for analyses. The ratio was followed by another jittered blank interval of 200, 300, or 400 ms. We then asked them to state whether the stocks belonged to themselves or to the other and whether they had chosen the stocks themselves or if they were chosen by the other. At the end of each trial, one of the following two questions, “Who chose the stock (A1),” or “Who owned the stock (A1)” appeared as the “question” screen. The two answer options: the “self” and the “other” (in words), were randomly presented on the left or right side of the “question” screen. The participants were asked to judge the owner or the chooser of the stock by pressing a corresponding key (n, m) as quickly as possible within 2 s. The “question” screen did not disappear until participants responded. The last screen in the trial is a blank and lasted for 500 ms (see Figure 1 for procedural details).

The participant was seated comfortably about 1 m in front of a computer screen in a dimly lit and electromagnetically shielded room. The experiment was administered on an Intel(R) Core(TM) i7-3770 CPU computer with a Del 24-in. CRT display, using Presentation software (Neurobehavioral System Inc.) to control the presentation and timing of stimuli. Participants completed a 5-min training session prior to the commencement of the main experiment. After that, each participant received 4 blocks of 696 trials, with each of the 2 (stock chooser: self, other) × 2(stock owner: self, other) × 2(stock outcome: rise, fall) 8 experimental conditions concludes 87 trials (39 trials for 3 and 9% ratio and 9 trials for 6%). We introduced this design with various stock price change ratios to simulate realistic stock market fluctuations as well as avoiding subjects' desensitization to experimental stimuli. The order of the trials was counterbalanced across 4 blocks using M-sequences (Buracas and Boynton, 2002). These are pseudo random sequences that have the advantage of being perfectly counterbalanced n trials back, so that each type of trial was preceded and followed equally frequently by all 8 types of trials, including itself. Because M-sequences have the advantage that each type of trial will be preceded and followed equally frequently by all types of trials, the negligible history effect is removed by this averaging procedure (Buracas and Boynton, 2002). Each of the eight conditions contained dozens of trials, and the brain responses were averaged by those trials for each condition. In addition, there were 48 trials in which the stocks were not preceded by any ratios. These trials were used as fillers to control for possible response strategies. The 696 trials were randomly sequenced for each participant. Participants were provided with a 5-min break mid-session between 4 blocks.

After the main experiment task, participants completed a 40-item mini-marker scale of Big-Five Personality trait (Saucier, 1994), which comprises five factors (extraversion, agreeableness, conscientiousness, emotional stability, and openness). This is a widely used measure of Big-Five Personality trait, particularly because it is short and can be easily used in experimental settings. The Cronbach's α of the Big-Five personality trait test ranged from 0.79 to 0.90 of the five factors sub-scale in this study. We used the five personality traits to investigate the relationship between behavioral and neural evidence. Participants also completed a 7-item perspective-taking questionnaire taken from Davis's subscale of interpersonal reactivity index (IRI) (Davis, 1980). The Cronbach's α was 0.68 in this study.

### EEG data acquisition

EEGs were recorded from 32 scalp sites using tin electrodes mounted in an elastic cap (Brain Products, Munich, Germany) according to the international 10–20 systems. The vertical electrooculogram (VEOG) was recorded supra-orbitally from the right eye. The horizontal EOG (HEOG) was recorded from electrodes placed at the outer canthus of left eye. All EEGs and EOGs were referenced online to an external electrode that was placed on the tip of nose and were re-referenced offline to the mean of the left and right mastoids. All electrode impedance was kept below 5 kΩ. The bio-signals were amplified with a band pass from 0.016 to 100 Hz and digitized on-line with a sampling frequency of 1000 Hz. EEG epochs of 1200 ms (with a 200-ms pre-stimulus baseline) were extracted offline for ERPs time-locked to the onset of the ratio of the stocks. Ocular artifacts were corrected with an eye-movement correction algorithm, which employs a regression analysis in combination with artifact averaging (Semlitsch et al., 1986).

### EEG data analysis

The EEG and EOG data were analyzed off-line using the Brain Vision Analyzer Software Package (Brain Products, Munich, Germany). All data were re-referenced off-line to the mean of the left and right mastoids. Ocular artifacts were corrected with an eye-movement correction algorithm (Semlitsch et al., 1986). The signal was filtered off-line using a band-pass of 0.1–30 Hz. Artifacts with potentials exceeding ±80 μV was rejected. Subsequently, epochs that ranged from −200 to +800 ms relative to the ratio of stock onset were extracted and normalized (by subtraction) to a 200 ms pre-cue onset baseline.

**Figure 2:**
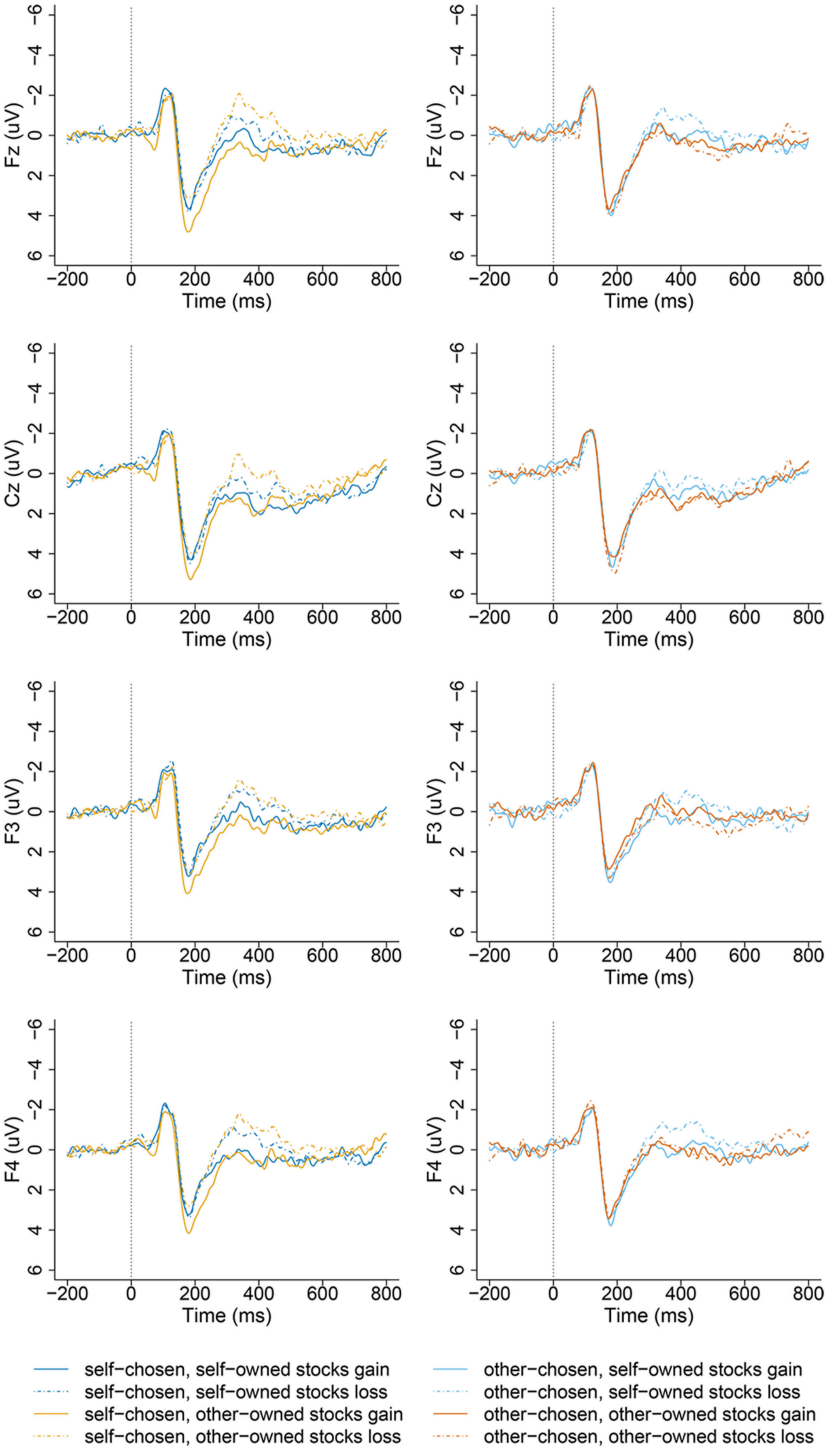
**Grand-averaged ERP waveforms from channel Fz, Cz, F3, F4 at the middle-frontal area as a function of stock types when playing different financial roles (self-chosen-self-owned, self-chosen-other-owned, other-chosen-self-owned, other-chosen-other-owned) and valence (gains and losses) of feedback outcomes, time-locked to the onset of stocks' outcome in the self-chosen group (left panel), and other-chosen group (right panel)**.

The ERP component analyzed in the current experiment is the FRN. Based on visual inspection of the ERP waveforms (Figure 2 shows FRN on sites F3, F4, Cz, Fz), mean amplitudes for the ERPs in the 320–420 ms window (FRN) were computed for the 8 experimental conditions, with each condition having on average 80–83 trails (ranging from 72 to 87 trials for each participant). The EEG epochs were averaged separately for 2 (stock chooser: self, other) × 2 (stock owner: self, other) × 2 (stock outcome: gain, loss) conditions (SCSO gain, SCSO loss, SCOO gain, SCOO loss, OCSO gain, OCSO loss, OCOO gain, and OCOO loss).

Additionally, to minimize the overlap of FRN with negative ERP components, we calculated the difference of FRN waveforms by subtracting the ERPs elicited by the gain trials from the ERPs elicited by loss trials in the 340–420 ms time window, following the methods of Holroyd and Krigolson (2007). We refer to the FRN difference effect between loss and gain as d-FRN. We calculated d-FRN for each of the four kinds of stocks separately when participants assumed each financial role: 2 (stock chooser: self, other) × 2 (stock owner: self, other).

This FRN difference effects (d-FRN) was defined as the mean value of the most negative component distributed on the anterior scalp over 320–420 ms in the experiments. We selected 10 electrodes of F3, F4, FC1, FC2, Fz, Cz, C3, C4, CP1, and CP2 in frontal area for FRN and d-FRN in statistical analysis (see Figure 2). These electrodes were chosen based on previous research that found FRN to be maximized at the fronto-central midline (Yeung et al., 2005; Hewig et al., 2007; Jia et al., 2007; Li et al., 2009). The average amplitude measure was adopted because it can reduce noise fluctuations compared with the base-to-peak approach, which is relatively insensitive to positive deflections in the time range of the FRN (Yeung et al., 2005; Cohen and Ranganath, 2007).

A three way repeated measures of analysis of variance (ANOVA) on the amplitude of d-FRN component was conducted with stock chooser (self, other), stock owner (self, other), and electrode position (10 electrodes: F3, F4, FC1, FC2, Fz, Cz, C3, C4, CP1, and CP2).

In addition, we averaged ERPs on the above-mentioned 10 electrodes in conjuction, taking the 10 electrodes as the anterior scalp area. A 2 × 2 × 2 mixed ANOVA on the selected 10 electrodes for FRN was performed, the three factors were: stock chooser (self, other), stock owner (self, other), and valence (gain, loss).

The computer program SPSS (version 20.0) was used. The Greenhouse–Geisser correction for violation of the assumption of sphericity was applied where appropriate.

## Results

### Behavioral results

Trials in which the participants did not respond within 2 s or responded incorrectly and trials in which the reaction times (RTs) exceeded three standard deviations from the mean in each experimental condition were excluded from data analysis. Approximately 1.00% of the total data points were lost due to these exclusions.

**Figure 3:**
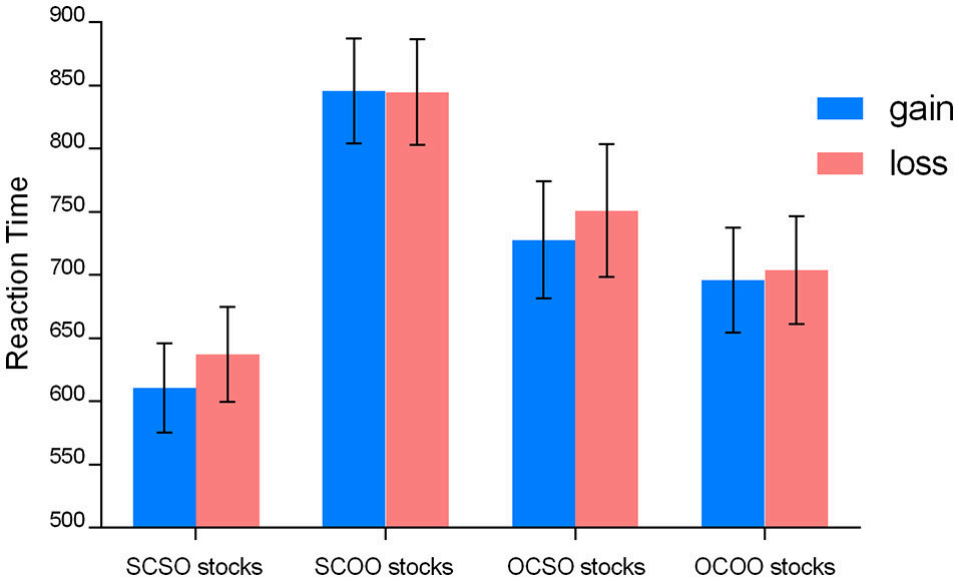
**Means of the RTs to the stock outcome (gains and losses) when playing the four different financial roles**. The error bars denote 1 SE across subjects for each condition.

We conducted a 2 (stock chooser: self, other) × 2 (stock owner: self, other) × 2 (stock outcome: gain, loss) mixed ANOVA on the RTs. The means of RTs showed in Figure 3. The ANOVA table for RT results is shown in Table 1.

**Table 1:** **The ANOVA for RT results**.

	***F***	***p***	**η^2^ partial**
Choosership	3.195	0.088	0.132
Ownership	15.908	0.001	0.431
Outcome (gain, loss)	10.482	0.004	0.333
Choosership ^*^ ownership	14.079	0.001	0.401
Choosership ^*^ outcome	0.096	0.76	0.005
Ownership ^*^ outcome	4.526	0.045	0.177
Choosership ^*^ ownership^*^ outcome	0.427	0.521	0.020

Results revealed a significant main effect of ownership, *F*_(1, 21)_ = 15.91, *p* = 0.001, η^2^ partial = 0.431, suggesting that the responses to self-owned stocks (mean ± SE, 681.64 ± 39.82 ms) were significantly faster than responses to other-owned stocks (772.43 ± 37.07 ms). Additionally, there was a reliable main effect of stock outcome, *F*_(1, 21)_ = 10.48, *p* = 0.004, η^2^ partial = 0.333, with the responses to the gain outcome (mean ± SE, 719.93 ± 35.84 ms) significantly faster than that to the loss outcome (mean ± SE, 734.14 ± 37.76 ms). There was no significant main effect of stock chooser and no interaction effect among the three factors (stock chooser, stock owner, and stock outcome). Although, the behavioral responses lagged behind the presentation of the stimuli in our paradigm, this finding is explained by the ownership effect and is consistent with previous studies showing that individuals generally respond faster to self-related items such as one's own name, phone number, or face photos (Greenwald and Farnham, 2000; Ma and Han, 2010). The negative stimuli representing a monetary loss (stock fall) attracted participants' attention and made participants respond slower, and it was more difficult for individuals to shift their attention to the next stimuli responses for the stock-owner/chooser judgment. This finding was consistent with the argument of increased expectancy, as the delayed reaction time may reflect the detection of conflict between expectancy and the actual outcome, irrespective of what attribute the expectancy is built upon (e.g., Wu and Zhou, 2009).

### ERP results

Based on visual inspection of the ERP waveforms (Figure 2), we analyzed the mean amplitude of the 320–420 ms time window after the onset of the stock outcomes. Based on previous research that found FRN to be maximized at the fronto-central midline (Yeung et al., 2005; Hewig et al., 2007; Jia et al., 2007; Li et al., 2009), we selected 10 electrodes, F3, F4, FC1, FC2, Fz, Cz, C3, C4, CP1, and CP2, in the frontal area for the FRN and d-FRN for assessment in the statistical analysis (see Figure 2).

**Figure 4:**
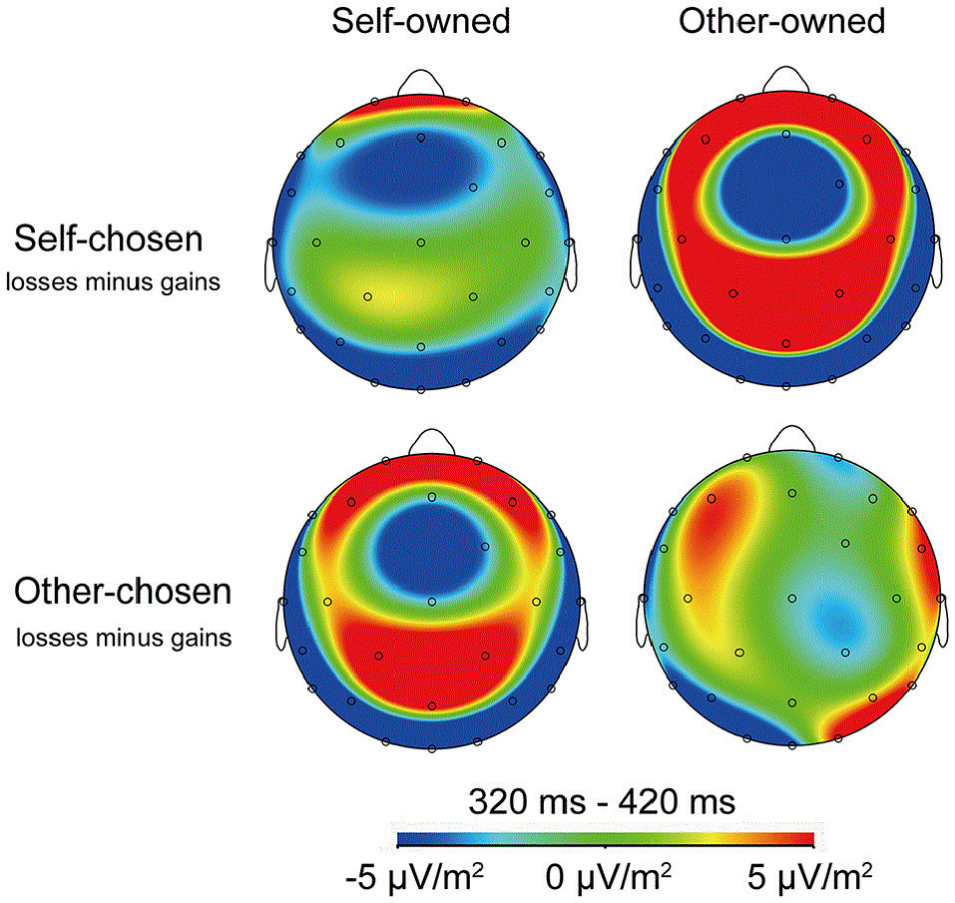
**Topographic maps for the sustained negativity of d-FRN**. FRN topography in response to the stock outcomes averaged over all subjects when playing four financial roles. Prefrontal lobe electrodes, including F3, F4, FC1, FC2, Fz, Cz, C3, C4, CP1, and CP2, had the largest FRN negative-going amplitudes.

It is clear from Figures 2, 4 that the choosership (self, other) altered the differential effect of the FRN between the ERP responses to the loss vs. gain outcomes of other-owned stocks but not for the self-owned stocks. Detailed statistical analyses confirmed this observation.

ANOVA with stock chooser (self, other), stock owner (self, other), valence of the stock outcomes (rise, fall), and the electrode (F3, F4, FC1, FC2, Fz, Cz, C3, C4, CP1, and CP2) yielded a significant three-way interaction between the stock chooser, ownership of the stocks, and the valence of the outcome, *F*_(1, 21)_ = 14.78, *p* = 0.001, η^2^ partial = 0.413. The four-way interaction between the stock chooser, ownership of the stocks, the valence of the outcome, and the electrode was not significant, *F*_(1, 21)_ = 1.74, *p* = 0.147, η^2^ partial = 0.077.

Then we performed analysis for the self-chosen stocks (SC) and other-chosen stocks (OC) separately. For the SC stocks, a 2 (stock owner: self, other) × 2 (stock outcome: gain, loss) × 10 (electrode: F3, F4, FC1, FC2, Fz, Cz, C3, C4, CP1, and CP2) repeated ANOVA revealed a significant main effect of valence (stock outcome), which indicated that the mean amplitude of the FRN across loss trials (0.61 ± 2.34 μV) had a more negative-going than across gain trials (2.25 ± 2.35 μV), *F*_(1, 21)_ = 22.00, *p* < 0.001, η^2^ partial = 0.512. Additionally, a two-way interaction between ownership and valence (stock outcome) approached significance, *F*_(1, 21)_ = 13.68, *p* = 0.001, η^2^ partial = 0.394, suggesting that the gain–loss effect was inconsistent between the two different owners (self, other). That is, the pattern of brain responses to a gain vs. loss outcome of self-chosen stocks was modulated by ownership. The electrode did not significantly interact with ownership [*F*_(1, 21)_ < 2.00].

**Figure 5:**
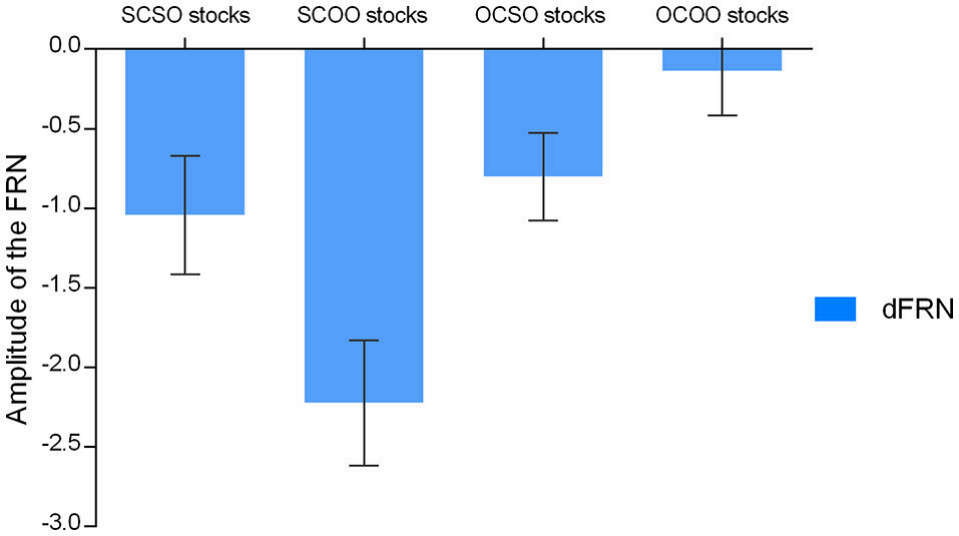
**Means of the amplitude of the FRN of the selected electrodes for ERP statistics of the stock outcome (gains and losses) when playing the four different financial roles**. The error bars denote 1 SE across subjects for each condition.

Simple tests were conducted for each of the two owners. For the SCSO stocks, the loss outcome (1.04 ± 2.45 μV) evoked more negative-going responses than the gain outcome (2.09 ± 2.32 μV), *F*_(1, 21)_ = 7.80, *p* = 0.011, η^2^ partial = 0.271. This is consistent with previous findings that reflect the violation of expectation to outcomes. Importantly, for the SCOO stocks, this pattern was also significant, such that the loss outcome (0.18 ± 2.23 μV) evoked more negative-going responses than the gain outcome (2.41 ± 2.57 μV), *F*_(1, 21)_ = 32.15, *p* < 0.001, η^2^ partial = 0. 605 (see Figures 2, 5).

**Figure 6:**
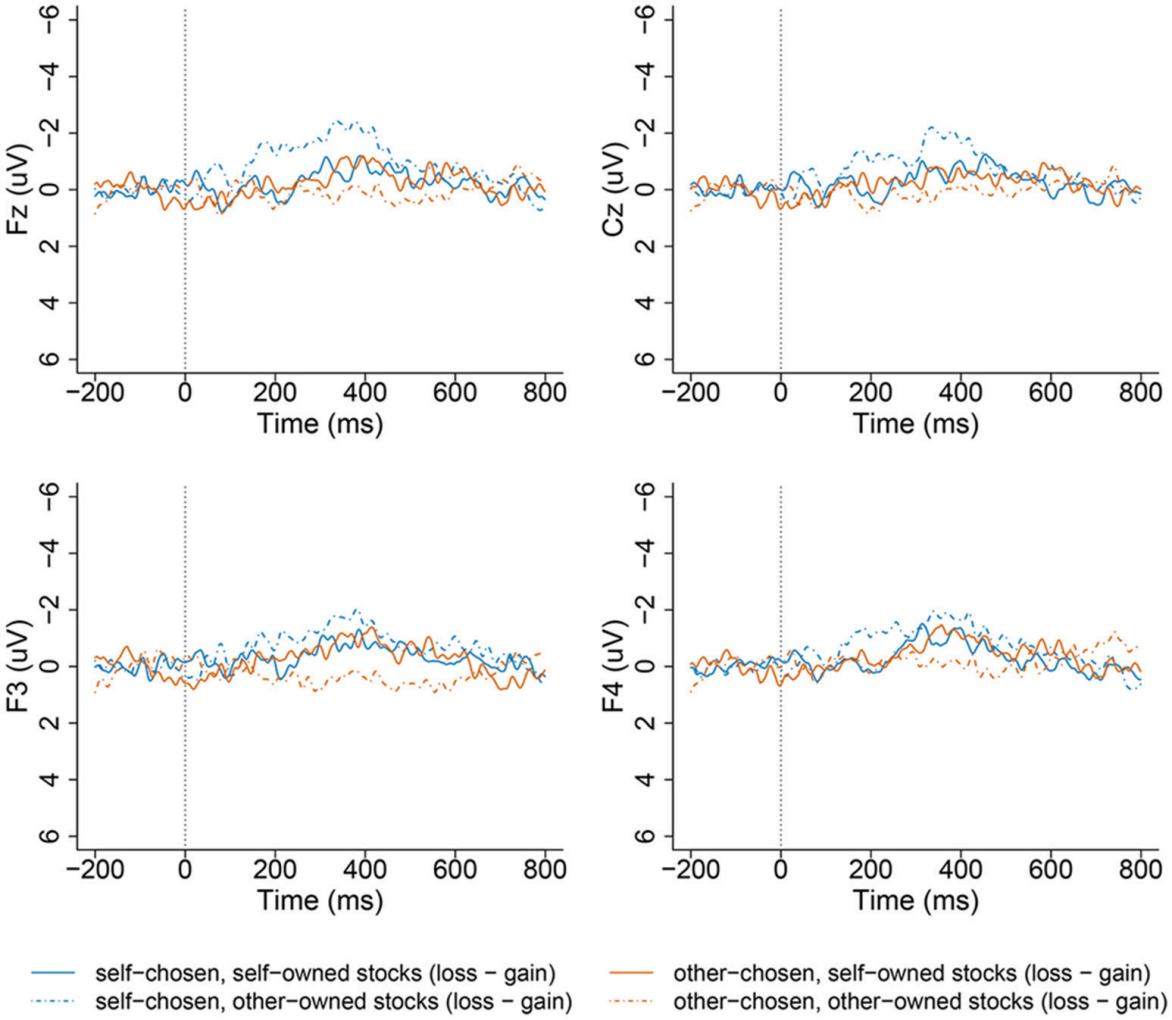
**The FRN difference waveform comparison among conditions playing different financial roles from channel Fz, Cz, F3, F4**.

**Figure 7:**
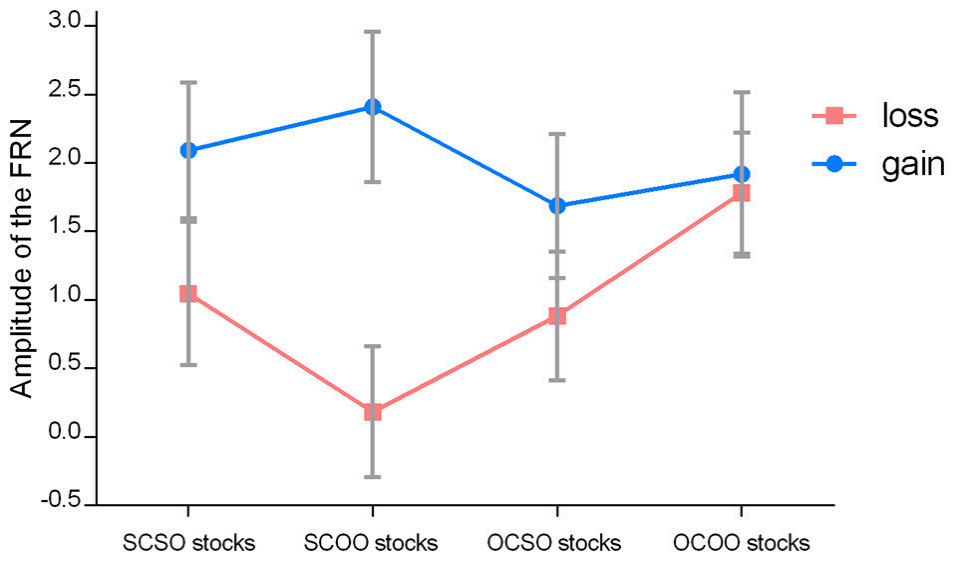
**Mean d-FRN amplitudes of the selected electrodes for ERP statistics for each of the conditions when playing the four different financial roles**. The error bars denote 1 SE across subjects for each condition.

Moreover, we focused on FRN difference (d-FRN) waves calculated by the brain responses evoked by the falling stock minus the brain responses evoked by the rising stock. For the difference of the waves of the FRN amplitude in SC stocks, a 2 (stock owner: self, other) × 10 (electrode: F3, F4, FC1, FC2, Fz, Cz, C3, C4, CP1, and CP2) ANOVA revealed that the main effect of stock owner was significant, *F*_(1, 21)_ = 13.68, *p* = 0.001, η^2^ partial = 0.528, such that the d-FRN to the SCOO stocks (−2.23±1.84 μV) was marginally larger compared to the SCSO stocks (−1.04±1.75 μV). The interaction between ownership and the electrode did not reach statistical significance, *F*_(1, 21)_ < 1.00 (see Figures 6, 7).

For the OC stocks, a 2 (stock owner: self, other) × 2 (stock outcome: gain, loss) × 10 (electrode: F3, F4, FC1, FC2, Fz, Cz, C3, C4, CP1, and CP2) repeated ANOVA was conducted, The main effect of valence (stock outcome) was significant, *F*_(1, 21)_ = 4.78, *p* = 0.04, η^2^ partial = 0.186. No other interaction was significant [*F*_(1, 21)_ < 2.3].

A *post-hoc* pairwise comparison with Bonferroni correction showed that the FRN amplitude difference between the gain and loss outcomes of OCSO stocks was significant, *F*_(1, 21)_ = 8.55, *p* = 0.008, η^2^ partial = 0.289, the loss outcome (0.88 ± 2.19 μV) evoked more negative-going responses than the gain outcome (1.69 ± 2.46 μV). While the difference between the gain and loss outcomes of OCOO stocks was not significant [*F*_(1, 21)_ = 0.24, *p* = 0.63; see Figures 2, 5].

In terms of the d-FRN's statistical analysis from the other-chosen stocks, a 2 (stock owner: self, other) × 10 (electrode: F3, F4, FC1, FC2, Fz, Cz, C3, C4, CP1, and CP2) ANOVA produced a marginally significant main effect of ownership, *F*_(1, 21)_ = 3.65, *p* = 0.07, η^2^ partial = 0.148. The d-FRN to the OCSO stocks (0.88 ± 2.19 μV) was more negative-going than the d-FRN to the OCOO stocks (1.69 ± 2.46 μV). Neither the main effect of the electrode nor the interaction between ownership and the electrode reached statistical significance, both *p* > 0.05 (see Figures 6, 7).

The second part of analysis was conducted for the brain responses to the self-owned (SO) and other-owned stocks (OO) separately.

For the SO stocks, a 2 (stock chooser: self, other) × 2 (stock outcome: gain, loss) × 10 (electrode: F3, F4, FC1, FC2, Fz, Cz, C3, C4, CP1, and CP2) repeated ANOVA revealed a significant main effect of valence (stock outcome), which indicated that the mean amplitude of the FRN across loss trials (0.96 ± 2.32 μV) was more negative-going than that across gain trials (1.88 ± 2.39 μV), *F*_(1, 21)_ = 10.65, *p* = 0.04, η^2^ partial = 0.337. The results did not demonstrate the main effect of the stock chooser, *F*_(1, 21)_ = 1.06, *p* = 0.31, η^2^ partial = 0.048. No other interaction was significant [*F*_(1, 21)_ < 1.5].

For the d-FRN amplitude of self-owned stocks (SO), an ANOVA of 2 (stock chooser: self, other) × 10 (electrode: F3, F4, FC1, FC2, Fz, Cz, C3, C4, CP1, and CP2) revealed that the main effect of the stock chooser was not significant, *F*_(1, 21)_ = 0.52, *p* = 0.48, η^2^ partial = 0.024. Neither the main effect of the electrode nor the interaction between the stock chooser and the electrode reached statistical significance, both *F*_(1, 21)_ < 1.00 (see Figures 6, 7).

For the other-owned stocks (OO), 2 (stock chooser: self, other) × 2 (stock outcome: gain, loss) × 10 (electrode: F3, F4, FC1, FC2, Fz, Cz, C3, C4, CP1, and CP2) repeated ANOVA revealed a significant main effect of valence, which indicated that the mean amplitude of the FRN across the loss trials (0.98 ± 2.15 μV) was more negative-going than across the gain trials (2.16 ± 2.69 μV), *F*_(1, 21)_ = 20.50, *p* < 0.001, η^2^ partial = 0.494. The main effect of the stock chooser was marginally significant, *F*_(1, 21)_ = 4.219, *p* = 0.053, η^2^ partial = 0.167. The two-way interaction between stock chooser and valence was significant, *F*_(1, 21)_ = 22.78, *p* < 0.001, η^2^ partial = 0.52. The electrode did not significantly interact with any of the experimental variables [*F*_(1, 21)_ < 2.2].

In terms of the d-FRN's statistical analysis with OO stocks, an ANOVA of 2 (stock chooser: self, other) × 10 (electrode: F3, F4, FC1, FC2, Fz, Cz, C3, C4, CP1, and CP2) showed a significant main effect of the stock chooser, *F*_(1, 21)_ = 22.78, *p* < 0.001, η^2^ partial = 0.52. Neither the main effect of the electrode nor the interaction between the stock chooser and the electrode reached statistical significance, both *p* > 0.05 (see Figures 6, 7).

The third part of the analysis analyzed FRN for gains and also for losses separately. We averaged the above 10 sites and conducted a two way ANOVA of 2 (stock chooser: self, other) × 2 (stock owner: self, other) on the FRN evoked by stock increase. No significant effect was found, *ps* > 0.05 (see Table 2).

**Table 2:** **The ANOVA for FRN evoked by stock increase**.

	***F***	***p***	**η^2^ partial**
Choosership	3.342	0.082	0.137
Ownership	1.534	0.229	0.068
Choosership ^*^ ownership	0.025	0.875	0.001

As the simple tests shown in Table 3 reveal, the FRN to SCSO stocks increase (2.08 ± 2.32 μV) was not significantly different from that to SCOO stocks (2.40 ± 2.57 μV); the FRN to OCSO stocks increase (1.68 ± 2.46 μV) was not significantly different from that to OCOO stocks increase (1.91 ± 2.81 μV); the FRN to SCSO stocks increase (2.08 ± 2.32 μV) was not significantly different from that to OCSO stocks increase (1.68 ± 2.46 μV); the FRN to SCOO stocks increase (2.40 ± 2.57 μV) was not significantly different from that to OCOO stocks increase (1.91 ± 2.81 μV).

**Table 3:** **Simple tests for FRN evoked by stock increase**.

	***F***	***p***	**η^2^ partial**
SCSO vs. SCOO	1.29	0.26	0.058
OCSO vs. OCOO	0.30	0.585	0.014
SCSO vs. OCSO	1.78	0.197	0.078
SCOO vs. OCOO	1.33	0.262	0.06

A two way ANOVA of 2 (stock chooser: self, other) × 2 (stock owner: self, other) on the FRN evoked by stock decrease showed that a main effect of choosership was significant (shown in Table 4). The FRN evoked by self-chosen stocks decrease (0.614 ± 2.34 μV) was more negative going than other-chosen stocks decrease (1.331 ± 2.13 μV). The interaction of choosership and ownership was significant (showed in Table 4). Simple tests showed that SCOO stocks decrease evoked more negative-going FRN (0.183 ± 2.23 μV) than SCSO stocks decrease evoked (1.045 ± 1.75 μV) in a significant level (showed as SCSO vs. SCOO in Table 5). OCSO stocks decrease evoked more negative-going FRN (0.882 ± 2.19 μV) than OCOO stocks decrease evoked (1.779 ± 2.08 μV) in a significant level (showed as OCSO vs. OCOO in Table 5).

**Table 4:** **The ANOVA for FRN evoked by stock decrease**.

	***F***	***p***	**η^2^ partial**
Choosership	18.57	0.000	0.469
Ownership	0.024	0.879	0.001
Choosership ^*^ ownership	13.20	0.002	0.386

**Table 5:** **Simple tests for FRN evoked by stock decrease**.

	***F***	***p***	**η^2^ partial**
SCSO vs. SCOO	12.24	0.002	0.368
OCSO vs. OCOO	9.97	0.005	0.322
SCSO vs. OCSO	0.234	0.634	0.011
SCOO vs. OCOO	43.58	0.000	0.675

The FRN evoked by SCSO stocks decrease (1.045 ± 1.75 μV) was not significantly different from that by OCSO stocks decrease (0.882 ± 2.19 μV) (showed as SCSO vs. OCSO in Table 5). SCOO stocks decrease evoked more negative-going FRN (0.183 ± 2.23 μV) than OCOO stocks decrease (1.779 ± 2.08 μV) at a significant level (showed as SCOO vs. OCOO in Table 5).

### Subjective rating and association with the ERPs

We collected post-recording questionnaires and performed a correlation analysis between the d-FRN amplitude and the big-five personality factors (Extraversion, Agreeableness, Conscientiousness, Emotional stability, and Openness). The coefficient alpha was 0.79 for Extraversion of the Big Five Personality, 0.88 for Agreeableness, 0.86 for Conscientiousness, 0.90 for Emotional Stability, and 0.83 for Openness.

A reliable negative correlation was observed between the difference waves of the FRN elicited by SCOO stocks and the participants' subjective rating of conscientiousness from the Five-Factor Model, *r* = −0.482, *p* = 0.023. However, the scores on the trait of conscientiousness showed no significant correlation with the d-FRN evoked by the SCSO stocks (*r* = 0.147, *p* = 0.513), the d-FRN evoked by OCSO stocks (*r* = −0.149, *p* = 0.507), and the d-FRN evoked by OCOO stocks (*r* = −0.332, *p* = 0.132). Those results showed that people with higher levels of conscientiousness had a greater elicited FRN discrepancy between losses and gains from SCOO stocks, reflecting their sensitivity to others' losses and gains. No other significant correlations were found among the other four personality traits and the d-FRN amplitude in the four person-stock relationships. Additionally, the correlations between the perspective-taking scores and the d-FRN amplitude did not reach a significant level for the SCSO stocks (*r* = −0.027, *p* = 0.906), the SCOO stocks (*r* = −0.162, *p* = −0.472), the OCSO stocks (*r* = 0.046, *p* = 0.839), and the OCOO stocks (*r* = 0.204, *p* = 0.303).

## Discussion

Using a new experimental paradigm, we manipulated four person-stock financial relationships to investigate the individual and interactive influences of perceived ownership and perceived choosership on stock outcome evaluation, which were reflected by neural responses. The results revealed that the ERP component FRN varied when individuals assumed different financial roles. This provides a more comprehensive picture of how our evaluations function when facing gains and losses belonging to ourselves and to others in large-scale financial markets. By doing so, we can explore the feature of human social emotions in monetary activities.

Results of response time analyses showed that the responses to gains were significantly faster than responses to losses. Results of brain potentials showed that FRN elicited by stock losses was larger than that elicited by stock gains. These results indicated that compared with stock gains, stocks losses seem to be of expectation violation which evoked more salient FRN at the brain level and increased peoples' reaction times at the behavioral level. Despite this relationship between the overall patterns in the behavioral and the brain responses to losses and gains, the brain responses were not significantly different between self-owned stocks (SO) and other-owned stocks (OO) while the reaction times were faster to SO than to OO stocks. However, in self-chosen stocks, people responded slower to SCOO stocks than SCSO stocks, and d-FRN was larger to SCOO stocks than SCSO stocks. Hence, FRN revealed sensitivity to losses and gains, while reaction time showed sensitivity to the ownership (SO vs. OO).

### The FRN effects in the four comparison group

#### Reversed ownership effect in self-chosen stocks: comparison between SCSO vs. SCOO

Electro-physiologically, participants displayed a statistically significant trend toward more negative FRN when observing losses than when observing gains of SCSO stocks. The trend indicated the presence of the FRN effect, that is, the d-FRN discrepancy (the difference wave between gains and losses). The reinforcement theory (Holroyd and Coles, 2002; Nieuwenhuis et al., 2004) suggests that the discrepancy between the actual outcomes and the expected outcomes signal the activity of a reinforcement learning system that continually evaluates ongoing events against the expected outcomes (Holroyd and Coles, 2002). The unexpected outcomes (stock decreases) elicited more significantly negative ERP responses than the expected outcomes (stock increases), reflecting the detection of an expectancy violation.

A surprising finding was that participants had more significantly negative FRN patterns when observing the losses than the gains of SCOO stocks, showing an expectancy violation for others' losses. This mimics the agent role. When assuming the role of an agent, the stock chooser (the agent) may show a guilty response to the stock owner's (the client's) losses. This result is in line with the hypothesis that FRN is a reflection of the motivational/affective impact of outcome events (Gehring and Willoughby, 2002; Yeung et al., 2005). Given that other-regarding responsibility induces guilt when a negative consequence is due to a fault of the self (Bell, 1982; Loomes and Sugden, 1982; Epstude and Roese, 2008; Yu and Zhou, 2009), it is possible that the chooser's conscientiousness trait induced a guilt response when stocks decreased.

The scenario of SCOO stocks was used to mimic the agent-client contract relationship. The economic actors (the agent and his/her client) develop contractual arrangements in the agency relationship, where each of the parties executes the legal obligations assigned to them. The agent takes charge of the transaction of the financial products for his/her client. Analogously, we argue that the participants' sense of responsibility to their clients was elicited when they were playing the agent role. In this scenario, guilt responses may be induced when seeing the client's loss. The correlation between questionnaire data and ERP data showed that trait *conscientiousness* of the chooser (the agent) was positively correlated with the d-FRN discrepancy elicited by the client's stocks, suggesting that participants high on conscientious were more likely to perceive clients' financial problems as unexpected negative events.

A similar study (Li et al., 2010) also demonstrated the effect of the personal responsibility on the outcome evaluative process in the self-regarding approach, with evidence that people high on personal responsibility had larger FRN effects for the SCOO outcomes in a gambling game.

Additionally, aside from the conscientiousness point of view, the reputation of one's decision-making may also affect the evaluation. The decision reputation of transactions for clients is very important for the agent, because clients may judge transaction results. According to the self-enhancement theory (McCrea and Hirt, 2001), people over-evaluate things related to themselves. For instance, they tend to think highly of their reputation and try to maintain a positive self-image in others' eyes (Beggan, 1992). As our findings indicate, the agents had positive expectations for the stock outcomes of their clients. Agents may feel ashamed when observing clients' losses because the losses can be seen as a threat to their own reputation.

Moreover, existing studies showed that the participant (the observer)'s FRN patterns reflected an empathic response to the other (the performer) in a social context (Fukushima and Hiraki, 2009; Ma et al., 2011), and the magnitude of the observer's FRN varied depending on the social distance between the performer and the observer (Fukushima and Hiraki, 2009). Our result is inconsistent with these previous findings. We found that people showed a larger FRN to those of a further social distance (SCOO) than those of a closer social distance (SCSO). Taken into consideration, the absence of significant correlations between perspective taking scores and the d-FRN amplitude of the SCOO stocks, we infer that the profit-based social distance between the agent and the client in the financial context is different from the familiarity between the “self” and the “other” in social contexts (e.g., friendship). This suggests that the “financial context” in our setting may not overlap with the “social context.”

Taken together, in the comparison Group One, people feel bad about both their own losses and others' losses. Although, humans are self-interested to some extent (Ma et al., 2011), they are also social creatures and exhibit consideration for others in social situations (Fehr and Camerer, 2007). This is consistent with the trend in neuroeconomics to refute the notion from traditional economics that people generally maximize their self-interest (Yeung and Sanfey, 2004; Hajcak et al., 2005, 2007; Sato et al., 2005; Toyomaki and Murohashi, 2005; Fehr and Camerer, 2007; Yu et al., 2007). Responsibility for others and self-related reputation may be two possible reasons for why people show other-regarding preferences in a financial context when the self is involved in the choosership. This argument is consistent with the literature in the effect of self-determination (Zuckerman et al., 1978).

As for evaluation comparison, ownership modulated the d-FRN effect. When participants assumed the buyer and agent roles, SCOO stock outcomes evoked a significantly larger d-FRN discrepancy than SCSO stocks outcomes, indicating that participants have more positive expectations toward other-owned outcomes than self-owned outcomes. In terms of unfavorable outcomes, a much higher expectancy violation to SCOO stocks losses than SCSO stocks losses was reflected by a larger FRN, while the FRN patterns elicited by gains were not significantly different. It seems that concern for others was stronger than self-regarding concerns when the chooser was the self. We infer that the other-regarding approach, which is induced by responsibility for others and self-related reputation, may be more dominant than the self-interested approach in a financial context. This intriguing finding was surprisingly contradictory to the ownership effect (Beggan, 1992), indicating a “*reversed ownership effect*,” which overshadowed the choosership effect.

We deduced that when participants confronted losses of their own and their clients, instead of adopting a selfish approach, the participants strengthened their responsibility toward the other and thus exhibited selfless behaviors, making them even more sensitive to the others' losses than to their own. If this is the case, interpersonal responsibility might be a more dominant feature of human beings than selfishness in the agency relationship, which in turn might ultimately be self-benefitting through the maintenance of a good reputation.

#### Ownership effect in other-chosen stocks: comparison between OCSO vs. OCOO

As for the comparison in the comparison Group Two, the results showed that ownership modulated the d-FRN effect: the d-FRN discrepancy was significant for the OCSO stock outcome evaluations but not for the OCOO outcome evaluations. When the chooser was the other, participants tended to have more positive expectations for self-owned stock outcomes than for other-owned stock outcomes, demonstrating the ownership effect (Beggan, 1992). It seems that people tend to prefer self-profit from stocks to non-self-profit regarding the stocks of others when they were not involved in the stock choosing process.

Participants exhibited no statistically significant FRN discrepancy between losses and gains when observing OCOO stock outcomes, indicating no sympathetic reactions toward strangers. This finding replicates results from previous neuro-economic laboratory studies that have revealed an indifferent attitude (i.e., no empathy) or even derogation of others when observing strangers' losses (Fukushima and Hiraki, 2006; Ma et al., 2011).

#### Choosership effect disappeared in the self-owned stocks: comparison between SCSO vs. OCSO

The d-FRN elicited by SCSO stock outcomes was not significantly different from that elicited by OCSO stock outcomes, suggesting that whether the chooser was the self or the other had little influence when the participant was the owner. This ERP data indicated that the choosership effect was overshadowed by the ownership effect. However, this finding is inconsistent with a previous object evaluation study (Huang et al., 2009), in which the choosership effect prevailed over the ownership effect on the subjects' preferences. A possible explanation for this inconsistent finding might be attribution. General objects, such as the cups or pens in Huang et al.'s (2009) study, were worth much less than financial products and referred to indirect monetary profit. It has been well-documented that people's evaluations would significantly vary across different amount of monetary values (Slonim and Roth, 1998; Wu and Zhou, 2009; Wei et al., 2015). For example, Slonim and Roth (1998) observed that players reduced the amount of their offers when the stakes were high in an ultimatum game but did not change their offers when the stakes were low. Such findings suggest that the quantity of outcomes plays some role in determining subjective responses to outcomes. As the outcome evaluation could be reflected in the FRN component, Wu and Zhou (2009), found that FRN is sensitive to the magnitude of reward feedback, with a larger d-FRN response to a larger reward than to a smaller reward. As objects are less valuable possessions, ownership does not affect object evaluation very much. In this condition, choosership could have an effect on object evaluations when objects are owned by the self. Different from evaluations of general objects, stocks, as a typical financial product, are closely related to monetary profit. Financial markets are quite different from routine items because the quantity of money involved in stock markets is very large. The outcome of stocks, even a small percentage of gain or loss, usually involves a large amount of monetary value change. People are thus more concerned about self-owned stocks' profits, regardless of whether they are self-chosen stocks or not. Given the relatively large monetary profit in the financial market, ownership tends to overshadow the choosership effect.

#### Choosership effect in the other-owned stocks: comparison between SCOO vs. OCOO

When the owner was the other, the self-chosen stock outcomes elicited a significantly larger d-FRN than the other-chosen stock outcomes, demonstrating a stronger positive expectation (see Figure 7). This implied a *choosership effect* even when participants do not own the stocks. Previous research on self-enhancement revealed that people overvalue self-related aspects (Kurman and Kurman, 2001). A choosership effect was induced by the overvaluation of the self-concept in self-chosen stocks. That is, individuals still prefer the stocks that they select when they do not own those stocks, and the choosership effect has a neural basis, as presented in the current literature.

### Implications and limitations

Our finding is the first to show that in a financial context, the agent's FRN is more negative toward a client's losses than toward the agent's own losses (see Figure 7), reflecting a higher expectation for self-chosen-other-owned stock outcomes. This provides a deeper understanding of the reinforcement theory that expectancy violation is affected by financial contexts. Although previous researchers have provided evidence to show that that people are not merely self-regarding in traditional economics (Yeung and Sanfey, 2004; Hajcak et al., 2005, 2007; Sato et al., 2005; Toyomaki and Murohashi, 2005), the comparison of the self-regarding and other-regarding approach has seldom been investigated. Our results provide direct comparison evidence that, when both the other-regarding and self-regarding approaches exist, humans may behave more altruistically than selfishly under particular circumstances where choosership and responsibility are involved.

The social distance between the “self” and the “other” was manipulated by different financial relationships in our paradigm. We decomposed the financial relationship into the owner role and the chooser role in the current study. Compared with previously used empirical economic situations (e.g., gamble games), our paradigm seems to be more relevant to the real world stock market that is the aggregation of buyers and sellers (a loose network of economic transactions, not a physical facility, or discrete entity) of stocks and imagined stock trade actions. The outcome of stocks, even a small percentage of gain or loss, usually involves a large amount of monetary value change, which is different from the relatively small amount of cash reward (e.g., several dollars or even cents) in traditional gambling games. The financial relationships between the “self” and the “other” in our study was assigned to anonymous unacquainted strangers and then primed by our experimental scenario. Participants in the simulated stock market were assigned different financial roles that included buyers of stocks for oneself (similar to those buying stocks for themselves and individual retail investors), buyers of stocks for others (similar to brokers), those who delegate others to buy stocks on their behalf (similar to those who buy funds), and those who simply observe others buying and selling stocks. We set up four roles according to perceived ownership and choosership, which helped to examine the neural foundation of complex human cognitive and emotional reactions to gains and losses in financial activities. This design is particularly useful for investigating responsibility, selfishness, and altruism in a more comparable setting.

The current study has a practical implication as well. This study mimics a financial agent in a real stock market. Results suggested the “agent” might feel more discomfort toward the client's loss than to the agent's own loss. This pattern of EEG/FRN results implies that people who work as stock brokers and fund managers, which, as high-paying careers, might be especially susceptible to tremendous pressure induced by the responsibility for others' outcomes. Our findings shed new light into the recruitment and training of these professionals.

The current study may have certain limitations that can be addressed in future research. First, although we measured the conscientious trait to represent responsibility, we only established the relationship between personality trait and d-FRN data. This is not a robust evidence to support that responsibility may result in a large d-FRN in the self-chosen-other-owned condition, and such causality needs to be further tested. Second, we did not directly measure individuals' self-reputation concerns. Third, we did not measure online emotional reactions related to the outcomes, such as guilt. Future research is needed to confirm the emotions that we speculated in this study. Fourth, we used an imaginary scenario and did not include actual stock investment actions. The ecological validity was limited and we did not have evidence to support our paradigm's validity. However, although we did not provide evidence that our paradigm was able to represent the large monetary scale of financial markets, it represents an important step to try to mimic the complicated financial market. Future research could examine the current findings among actual stockholders or fund managers in the real world to increase the external validity.

Additionally, although we used M-sequence to balance the sequence effect between all eight conditions, the sequence of ratios (3, 6, 9%) was presented randomly, which may induce a negligible history effect. We could not fully exclude the possible sequence effect of different ratios for sure. Finally, we did not separate different ratios of price change (3 vs. 9%) because the number of trials for each ratio was not enough for ERP analysis in our design. We only recorded the increase or decrease of price change. There may be a value size effect between 3 and 9%. Future studies could study the size effect on outcome evaluation in the four financial scenarios.

In conclusion, by recording participants' neural and behavioral reactions to stock outcomes, the present study investigated the neural mechanism of how financial contextual factors affect outcome evaluations, reflected by amplitudes of tFRN, in a stock observation task. We found a “reversed ownership effect” for the self-chosen stocks (comparison group One) and the ownership effect in other chosen stocks (comparison Group Two). The choosership effect disappeared under the influence of the perceived ownership (self-owned stocks, comparison Group Three), whereas the choosership effect was robust for other-owned stocks (comparison Group Four). Our findings refute the hypothesis from traditional economics that people only act to maximize their self-interest and provides concrete neural evidence showing that people are social creatures and can be other-regarding in financial situations (Fehr and Camerer, 2007). While we admit that people are self-interested to a certain extent, at least in certain contexts, altruism prevails over self-regard in the agency relationship. In addition, the research paradigm reported here provides a useful experimental pattern that helps to explain human nature by comparing the neural underpinnings of human financial behaviors. These results are particularly relevant to a new research area that we call *social neuro finance*.

## Ethics statement

Committee for Protecting Human and Animal Subjects, Department of Psychology, Peking University. The human participants were told to record their brain responses by EEG equipment which will not hurt to them. They were told there would be no any dangers while they were doing the experiment in which they would see some texts in a computer screen and react by push some buttons on the keyboard. They were told their rights and they can decide to or not to participate in this experiment, and they had the right to quit the experiment at any time of the experiment. They were reward for about 15 US dollars for their participation.

## Author contributions

LW proposed the main research idea; ZS and LW made the research design; ZS designed the experimental materials and ran the statistics; ZS and HW conducted the experiment; ZS and LW made the discussion and wrote the manuscript.

## Funding

NSFC Grant #71021001, #91224008, and #91324201; Beijing Positive Psychology Foundation Grant #0020344. It was also supported by Taetea Group.

### Conflict of interest statement

The authors declare that the research was conducted in the absence of any commercial or financial relationships that could be construed as a potential conflict of interest.
